# Prevalence of hearing loss and health vulnerability in children aged 25 to 36 months: an analysis of spatial distribution

**DOI:** 10.1590/2317-1782/20232021189en

**Published:** 2023-12-04

**Authors:** Aline Aparecida Lopes, Amélia Augusta de Lima Friche, Stela Maris Aguiar Lemos, Lorena Bicalho, Artur Marins Moreto Silva, Thamara Suzi dos Santos, Renata Cristina Cordeiro Diniz Oliveira, Paul Avan, Sirley Alves da Silva Carvalho

**Affiliations:** 1 Programa de Pós-graduação em Ciências Fonoaudiológicas, Universidade Federal de Minas Gerais - UFMG - Belo Horizonte (MG), Brasil.; 2 Departamento de Fonoaudiologia, Faculdade de Medicina, Universidade Federal de Minas Gerais - UFMG - Belo Horizonte (MG), Brasil.; 3 Programa de Pós-graduação em Ciências da Saúde da Criança e do Adolescente, Universidade Federal de Minas Gerais - UFMG - Belo Horizonte (MG), Brasil.; 4 Instituto de Geociências, Universidade Federal de Minas Gerais - UFMG - Belo Horizonte (MG), Brasil.; 5 Laboratoire de Biophysique Neurosensorielle, Université Clermont-Auvergne, France.; 6 Institut de l'Audition de l'Institut Pasteur - INSERM - Paris, France.

**Keywords:** Child Health, Hearing Loss, Health Vulnerability, Child, Preschool

## Abstract

**Purpose:**

To analyze the association between hearing loss and health vulnerability in children aged 25 to 36 months.

**Methods:**

Analytical observational cross-sectional study conducted through child hearing screening in nine day-care centers. The screening consisted of anamnesis, otoscopy, tympanometry, transient otoacoustic emissions, and pure tone audiometry. For each exam performed, the 'pass' and 'fail' criteria were established. The children's residential addresses were georeferenced and a choropleth map of the spatial distribution was built, considering the Health Vulnerability Index (HVI). The analysis of the association between the HVI and the variables sex, auditory assessment, and region area of the household was performed using Pearson's Chi-square and Fisher's Exact tests.

**Results:**

Ninety-five children of both sexes were evaluated, of which 44.7% presented alterations in at least one of the exams performed, being referred for otorhinolaryngological evaluation and subsequent auditory assessment. Of the observed changes, 36.9% occurred in the tympanometry and 7.8% in the transient otoacoustic emissions. Among children referred for reassessment, 9.7% were diagnosed with conductive hearing loss, 13.6% results within normal limits and 21.4% did not attend for assessment. Of the children who presented the final diagnosis of conductive hearing loss (9.7%), 1.9% were classified as low-risk HVI and 6.8% as medium-risk HVI. There was statistical significance between HVI and the child's place of residence.

**Conclusion:**

The association between hearing loss and HIV was not statistically significant; however, it was possible to observe that 77.7% of the children with hearing loss resided in sectors with medium- risk HIV

## INTRODUCTION

The concepts of hearing and vulnerability may seem subtly articulated. However, it is essential to discuss auditory diagnoses considering vulnerability and health conditions, especially in early childhood.

This discussion does not exclude the understanding that children’s first years of life is when the central auditory system undergoes its greatest maturation, and the auditory pathway presents greater neural plasticity^(1-3)^, as well as the importance of the access to and transit in the health system to ensure timely comprehensive assistance.

The identification and diagnosis process in children is complex and may be hindered by socioeconomic, assistance, and cultural barriers. Studies show that hearing loss is identified at the mean age of 2 and a half to 3 years, which is already late, given the scientific recommendations that electronic devices should be preferably fit before 6 months old^(3-6)^. According to previous research data despite being mandatory, the coverage of neonatal hearing screening (NHS) in the Southeast Region reaches only 70.3% of newborns^(7)^. The study shows that Brazilian rates are still low, despite their positive evolution - between January 2008 and June 2015, the final coverage was 31.8%, ranging between Brazilian regions from 19% to 100%. This indicates unequal spatial distribution, with better coverages concentrated in the South and Southeast Regions^(7)^.

In this context, it is unquestionably necessary to discuss vulnerability and the use of its indicators in hearing prevalence and diagnosis studies. The literature points out that using these instruments may help understand the needs and data to develop policies, make decisions, and publicize information^(8)^. This study considered vulnerability from the perspective of social health determinants, approaching the Health Vulnerability Index (HVI) developed by the Municipal Department of Health of Belo Horizonte^(9)^, articulating it with the hearing screening process in the age range from 25 to 36 months.

The initial diagnosis knowingly does not reach all Brazilian children, and many of them get to preschool without adequate approach. Thus, hearing screening in preschoolers may prevent difficulties in oral and written language development, as both are directly related to hearing. About 50% of hearing losses could be avoided or have their sequelae minimized if identification, diagnosis, and rehabilitation measures were taken earlier, especially in schoolchildren^(6,10)^.

Given the relevance of diagnosing hearing loss at an adequate age in childhood, its impact on children’s global development and quality of life, and its relationship with social determinants, this study aimed to analyze the association between hearing changes and health vulnerability in children aged 25 to 36 months attending public day care centers.

## METHODS

This cross-sectional, analytical, observational study was approved by the Ethics Committee of the Federal University of Minas Gerais under number 931.831 and is part of a larger study entitled Development of a Pediatric Hearing Screening Instrument.

The sample of the present study comprised children aged 25 to 36 months attending day care centers partnered with the municipal government of Belo Horizonte. The sample calculation was made for the larger project, which assessed children in three age ranges: 12 to 18 months, 25 to 36 months, and 37 to 48 months. This research assessed only those aged 25 to 36 months. The main study’s sample calculation addressed the three age groups. It considered a 5% error, a 5% level of significance, a 10% population loss, and that the actual hearing change rates in the population of Belo Horizonte would hardly exceed 30%. Thus, the final calculation suggested a sample of 108 in each age group. However, in the present study, only 95 parents/guardians of children aged 25 to 36 months signed an informed consent form (ICF), which defined the sample size.

This study encompassed nine day care centers in the metropolitan area of Belo Horizonte, located in each of the nine administrative regions of the city: Barreiro, Central-South, East, Northeast, Northwest, North, West, Pampulha, and Venda Nova. One day care center from each region was selected to ensure the whole municipality of Belo Horizonte was represented. Their principals were asked whether they agreed to participate, and the parents/guardians of all children in the study’s age range were invited to the research.

The inclusion criteria to participate in the study were children aged 25 to 36 months, attending day care centers partnered with the municipal government of Belo Horizonte, and whose parents/guardians agreed with their participation and signed an ICF. Children who did not attend the day care center on the day of screening or diagnostic assessment or whose parents informed they had given up on their participation at any stage of the study were excluded.

The study had three stages - the first one was named screening, the second one, diagnosis, and the last one, georeferencing. The screening was carried out at the day care centers, and the diagnosis (for those who “failed” in the first stage) was conducted at the HC/UFMG São Geraldo Hospital. Each stage had specific procedures.

The first-stage assessments took place between February and December 2017, in two weekdays, one of them in the morning and the other in the afternoon. The whole assessment took a mean of 10 to 20 minutes per child. The terms “pass” and “fail” were used to classify the audiological examination results. The following screening procedures were used:

- Otoscopy: the external auditory meatus was inspected with a Pocket Junior otoscope with fiber-optic light 2.5V 22840 - Welch Allyn.
- Tympanometry: conducted with Madsen Otoflex 100 acoustic-immittance meter, calibrated according to ANSI S3.6, to assess whether the tympanic-ossicular chain was intact with the tympanometry curve and research the ipsilateral and contralateral acoustic reflexes in all children assessed with otoscopy. Tympanometry results were analyzed based on the normal standard suggested by Jerger and Jerger^(11)^ .
- Transient otoacoustic emissions (TEOAE): performed in a portable sound booth, mini model, measuring 90 x 90 x 155 cm. TEOAE was recorded with Elios^®^ equipment, manufactured by ECHODIA. The record protocol in screening mode used nonlinear click stimuli at 80 dBSPL, with a 12-millisecond test window, totaling 512 stimuli. TEOAE were considered present when the reproducibility was equal to or greater than 70% and the signal-to-noise ratio (SNR) was equal to or greater than 3 dB, using the “pass/fail” criteria. Children whose unilateral or bilateral result was “fail” were referred for otorhinolaryngological and speech-language-hearing assessments.
  ● Pure-tone threshold audiometry: conducted in a portable sound booth, mini model, measuring 90 x 90 x 155 cm, using Elios^®^ equipment, manufactured by ECHODIA, and TDH supra-aural earphones. The air-conduction thresholds were researched (sweep technique at 20 dB), at frequencies: 500, 1000, 2000 and 4000 Hz. always using playful resources to entertain the child. Those whose auditory thresholds were above 20 dBHL in at least one of the test frequencies (i.e., “fail”) were referred for otorhinolaryngological and speech-language-hearing assessments.

The parents received a feedback document with the examinations and results. In the case of children who “failed” any examination, the document informed the scheduled appointment for otorhinolaryngological and speech-language-hearing assessment at the audiology service to reach a diagnosis.

The second stage - i.e., the diagnostic assessment of children who “failed” at least one of the examinations conducted at the day care center - took place at the HC/UFMG São Geraldo outpatient center. The diagnostic assessment had the following procedures: otorhinolaryngological assessment, tympanometry, TEOAE, conditioned pure-tone audiometry, and auditory brainstem response (ABR). The assessment team had an otorhinolaryngologist, two speech-language-hearing therapists, and two undergraduate speech-language-hearing interns. They conducted the following procedures:

- Otorhinolaryngological assessment: thoroughly conducted, removing cerumen with warm water and curette, when necessary. The cases with acute upper airway infection underwent treatment and were later referred to community health centers for follow-up with an outpatient otorhinolaryngologist. Chronic cases received instructions and were also referred to community health centers.
- Acoustic immittance: The equipment used for diagnosis was an acoustic-immittance meter, model AT 235, manufactured by Interacoustics, calibrated on August 23, 2017, with certificate no. 4251/2017. Ipsilateral and contralateral acoustic reflexes were researched at 1, 2, and 4 kHz. Tympanometry results were analyzed based on the normal standards suggested by Jerger and Jerger^(11)^.
- Pure-tone threshold audiometry and speech audiometry: conducted with an audiometer AD229b, manufactured by Interacoustics, calibrated on August 23, 2017, with certificate no. 4246/2017. It used the descending technique at 250, 500, 1000, 2000, 3000, 4000, and 8000 Hz (air-conduction) and 500, 1000, 2000, 3000, and 4000 Hz (bone-conduction). Speech audiometry was conducted in a simple order, using auditory masking when necessary. The results were analyzed based on the normal standard proposed by the International Bureau for Audiophonology (BIAP)^(12)^.
- ABR: recorded with Elios^®^ equipment, manufactured by ECHODIA, researching the electrophysiological thresholds and the integrity of the auditory pathways. The protocol used rarefied click stimuli, 3000-Hz low-pass filter, 50-Hz high-pass filter, with 17 clicks per second and at least 1000 acquisitions. The electrodes were positioned at Cz, Fz, A1, and A2, and the stimuli were presented via insert earphones. Impedance was maintained at the maximum limit of 5 kilohms. The integrity of the auditory pathways was assessed in two sweeps at 80 dBnHL, researching the latencies of waves I, III, and V, the interpeak intervals, and the reproducibility. The electrophysiological threshold was determined as the last intensity at which the wave V appeared.
  ● TEOAE: performed with Elios^®^ equipment, manufactured by ECHODIA, using nonlinear click stimuli at 80 dBSPL and 12-millisecond test window, totaling 512 stimuli. TEOAEs were considered present when the reproducibility was equal to or greater than 70% and SNR was equal to or greater than 3 dB.

As for the flow of attention in the second stage, after the otorhinolaryngological assessment and procedure, the children were referred for acoustic immittance and then audiometry and TEOAE. Those whose examinations were all within normal standards obtained the result of normal hearing. Children who could not be conditioned to undergo audiometry were referred for objective examination (ABR). At the end of the assessment, parents/guardians received the examination results. When necessary, the children were referred to the family’s reference community health center to enroll the child in the Hearing Health Service.

Lastly, data were treated and analyzed. The participating children’s home address was searched to identify their census sector and HVI, developed and used by the Municipal Department of Health of Belo Horizonte to organize the health services in the municipality. HVI is a compound indicator that uses 2010 census data^(13)^ and socioeconomic and health variables to analyze the characteristics of population groups who live in the various census sectors. The index comprises variables on permanent private homes with inadequate or absent water supply, sewage, and waste collection; the number of people per household; the percentage of illiterate people; the percentage of private homes with per capita income of up to half a minimum wage; the householder’s mean nominal monthly income; and the percentage of multiracial, black, or indigenous people. At the end of the process, HVI is classified into the following four categories: low, medium, high, and very high health vulnerability. This study identified the risk corresponding to the census sectors in which each participating child lived. In the analysis, the high and very high categories were grouped into one.

The following variables were selected for data analysis: sex; age (in months); region of residence; otoscopy, tympanometry, otoacoustic emissions (OAE), and audiometry screening results (classified as either “passed” or “failed”); referrals (yes or no); diagnostic assessment results (normal examinations or hearing loss); and HVI of the census sectors where the children lived (categorized in low, medium, and high/very high risk).

Georeferencing of the places where children lived was based on their home addresses, then identifying the respective census sectors and classifications, using ArcGis program, version 10.5, ArcMap tool, and Google Earth Pro, version 7.3.1. The children’s addresses were located with Google Earth Pro, from which .kml extension files were extracted for georeferencing on ArcGis, thus producing maps over the 2012-HVI basis of the municipal government of Belo Horizonte. Out of the total 95 addresses, 94.8% were processed and georeferenced, identifying their census sectors in Google Earth. Five addresses could not be manually located, which corresponds to 5.2% of all addresses available.

The descriptive analysis used the distribution of absolute and relative frequencies of the categorical variables and measures of central tendency, position, and dispersion of the children’s ages.

The association analysis considered two response variables - (1) OAE results and (2) HVI. It was assessed whether the former associated with audiometry and tympanometry results in the screening and diagnostic assessments and whether the latter associated with sex, examination results, referrals, and diagnoses. The association analyses used Peason’s chi-square and Fisher’s exact tests, considering statistically significant associations with p-value ≤ 0.05. Data were entered, processed, and analyzed in SPSS software, version 21.0.

## RESULTS

**Figure 1:**
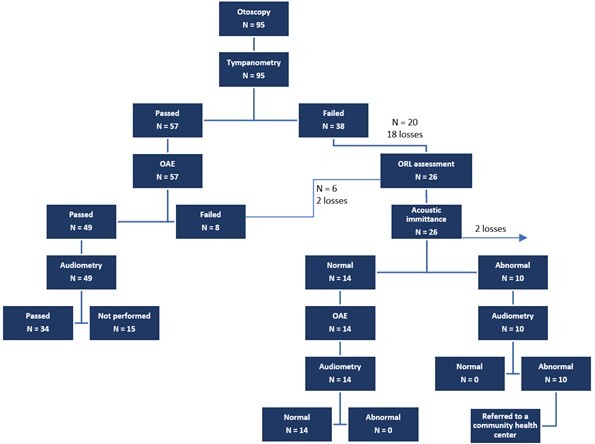
Flowchart of the research procedures protocol and participants distribution

The study assessed 95 children who attended nine day care centers located in the nine regions of Belo Horizonte. Of the 46 subjects referred for diagnosis, 26 attended the assessment. Of these, 14 had normal results at the end of the assessments; two did not let the professionals assess them and were submitted to ABR, obtaining normal results; and 10 had abnormal results. Hence, the result/diagnosis of 10.5% of the 26 subjects assessed was conductive hearing loss; they were referred to the community health center to be enrolled in the Hearing Health Service for follow-up. Participants who did not attend stage 2 (diagnosis) on the scheduled date were recontacted. However, 22 children (47.8%) did not attend it, even after various rescheduling attempts (Figure 1, Table 1).

**Table 1:** Distribution of the descriptive analysis regarding sex and auditory examinations

**Variables**	**N**	**%**
**Sex**		
Females	58	56.3
Males	45	43.7
Total	103	100.0
**Otoscopy**		
Normal	95	92.2
Did not allow examination	7	6.8
Did not attend	1	1.0
Total	103	100.0
**Tympanometry**		
Type A	57	60
Type B	36	37.9
Type C	2	2.1
Total	95	100.0
**Otoacoustic emissions**		
Failed	8	8.4
Passed	49	51.6
Not applicable	38	40
Total	95	100.0
**Audiometry**		
Failed	0	0.0
Passed	34	35.8
Did not allow examination	15	15.8
Not applicable	46	48.4
Total	95	100.0

**Caption:** N = number of individuals.

Most (56.3%) of the 95 children included in the research were females (Table 1), with a mean age of 29.9 months, median of 30.0, and standard deviation of 3.5. Regarding examinations in the screening stage, 92.2% of the study population had normal otoscopy, and 60% had type A tympanograms. Also, most of them “passed” the OAE examinations (51.6%) and audiometry (35.8%) (Table 1).

In screening, 46 children (i.e., 48.4% of the sample) failed and were, therefore, referred to the second stage. However, 22 of them did not attend the diagnostic assessment - i.e., only 52.2% were assessed. Hence, those who attended diagnostic assessment and had normal results in all examinations (tympanometry, OAE, and audiometry) were 14.7% of the total sample, whereas those with abnormal results were 10.5% (Table 2).

**Table 2:** Distribution of results in the diagnostic stage

Procedure after Stage 1	**N**	**%**
**Referral**		
No, normal examination results	49	51.5
Yes, abnormal examination results	46	48.4
Total	95	100.0
**Diagnostic assessment**		
Non-attendance	22	47.8
Normal examination results	14	30.4
Conductive loss	10	21.7
Total	46	100.0
Results in Stage 2 - Diagnostic assessment		
**Acoustic Immittance**		
Abnormal	10	10.5
Normal	14	14.7
Did not allow/Did not attend	22	23.1
Not applicable	49	51.6
Total	95	100.0
**OAE**		
Abnormal	10	10.5
Normal	14	14.7
Did not allow/Did not attend	22	23.1
Not applicable	49	51.6
Total	95	100.0
**Audiometry**		
Abnormal	10	10.5
Normal	14	14.7
Did not allow/Did not attend	22	23.1
Not applicable	49	51.6
Total	95	100.0

**Caption:** N = number of individuals.

**Figure 2:**
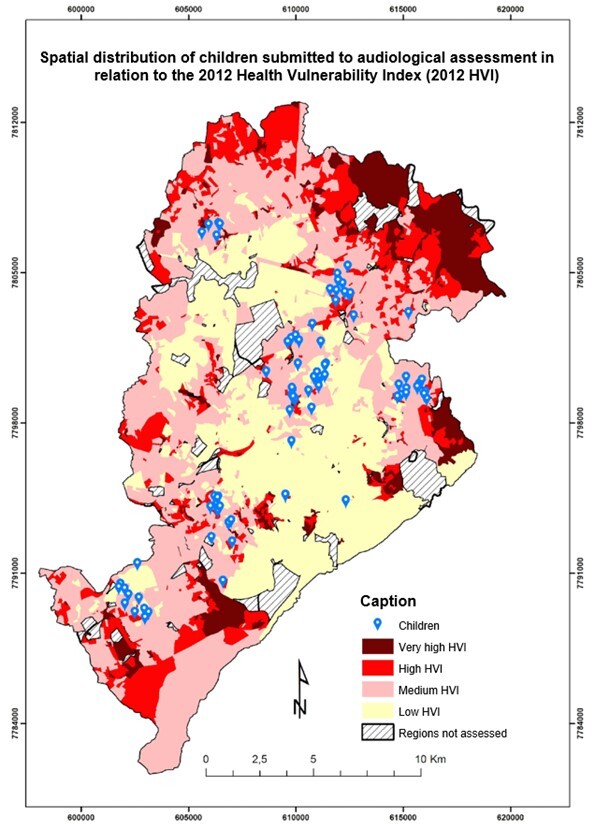
Map of the spatial distribution of children submitted to audiological assessment in relation to the 2012 Health Vulnerability Index

The 95 children whose parents signed an ICF were distributed into regions as follows: 12 lived in Barreiro; 10, in the Central-South; 16, in the East; 14, in the Northeast; 10, in the Northwest; 14, in the West; 10, in Pampulha; and 4, in Venda Nova. As for HVI, 59.2% of participating children lived in medium-risk census regions, while 9.7% lived in high/very high-risk census regions. It was not possible to identify the regions of 4.9% of the participants because they did not live in Belo Horizonte (Figure 2).

Of the children who failed the tympanometry in the first stage (screening), 10.0% lived in low-risk census regions; 24.4%, in medium-risk; and 4.4% in regions that posed a high/very high risk of acquiring a disease and dying. Of those who failed OAE, also performed at the day care centers, 3.6% lived in low-risk census regions; 9.1%, in medium-risk; and 1.8%, in high/very high-risk census regions.

The association analysis between TEOAE results in the second stage and tympanometry and audiometry results (Table 3) revealed that OAEs were statistically significantly associated with tympanometry and audiometry (p ≤ 0.001).

**Table 3:** Association between transient otoacoustic emission results and the other audiological diagnostic examinations

**Variables**	**Otoacoustic Emissions**			**p-value**
	**Failed** **N (%)**	**Passed** **N (%)**	**Total** **N (%)**	
**Acoustic Immittance**				
Abnormal	10 (41.7)	0 (0.0)	10 (41.7)	≤0.001
Normal	0 (0.0)	14 (58.3)	14 (58.3)	
Total	10 (41.7)	14 (58.3)	24 (100.0)	
**Audiometry**				
Abnormal	10 (41.7)	0 (0.0)	10 (41.7)	≤0.001
Normal	0 (0.0)	14 (58.3)	14 (58.3)	
Total	10 (41.7)	14 (58.3)	24 (100.0)	

Fisher’s exact test

**Caption:** N = number of individuals.

The responses “fail” and “does not live in Belo Horizonte” were excluded from the georeferencing for the association analysis between HVI and screening examinations. It revealed no statistically significant association between any of the results (Table 4). However, it was not possible to perform an association analysis with meatoscopy and audiometry for the lack of “fail” responses.

**Table 4:** Association between the Health Vulnerability Index and sex and audiological examinations

**Variables**	**Health Vulnerability Index**				**p-value**
	**Low**	**Medium**	**High/Very High**	**Total**	
	**N (%)**	**N (%)**	**N (%)**	**N (%)**	
**Sex**					
Females	11 (12.2)	36 (40.0)	6 (6.7)	53 (58.9)	0.993
Males	8 (8.9)	25 (27.8)	4 (4.4)	37 (41.1)	
Total	19 (21.1)	61 (67.8)	10 (11.1)	90 (100.0)	
**Otoscopy**					
Abnormal	0 (0.0)	0 (0.0)	0 (0.0)	0 (0.0)	----
Normal	19 (21.1)	61 (67.8)	10 (11.1)	90 (100.0)	
Total	19 (21.1)	61 (67.8)	10 (11.1)	90 (100.0)	
**Tympanometry**					
Failed	9 (10.0)	22 (24.4)	4 (4.4)	35 (38.9)	0.675
Passed	10 (11.1)	39 (43.3)	6 (6.7)	55 (66.1)	
Total	19 (21.1)	61 (67.8)	10 (11.1)	90 (100.0)	
**OAE**					
Failed	2 (3.6)	5 (9.1)	1 (1.8)	8 (14.5)	0.838
Passed	8 (14.5)	34 (61.8)	5 (9.1)	47 (85.5)	
Total	10 (18.2)	39 (70.9)	6 (10.9)	55 (100.0)	
**Audiometry**					
Failed	0 (0.0)	0 (0.0)	0 (0.0)	0 (0.0)	----
Passed	6 (18.2)	23 (69.7)	4 (12.1)	33 (100.0)	
Total	6 (18.2)	23 (69.7)	4 (12.1)	33 (100.0)	

Pearson’s chi-square test

**Caption:** N = number of individuals (varies according to the characteristics of the variable); OAE = otoacoustic emissions.

The association analysis between HVI and sex and second-stage audiological examinations found no statistically significant result in any of the analyses (Table 5).

**Table 5:** Association between the Health Vulnerability Index and sex, audiological diagnostic examinations, and procedures

**Variables**	**Health Vulnerability Index**				**p-value**
	**Low**	**Medium**	**High/Very High**	**Total**	
	**N (%)**	**N (%)**	**N (%)**	**N (%)**	
**Sex**					
Females	11 (12.2)	36 (40.0)	6 (6.7)	53 (58.9)	0.993
Males	8 (8.9)	25 (27.8)	4 (4.4)	37 (41.1)	
Total	19 (21.1)	61 (67.8)	10 (11.1)	90 (100.0)	
**Tympanometry**					
Failed	2 (8.7)	7 (30.4)	0 (0.0)	9 (39.1)	0.233
Passed	1 (4.3)	10 (43.5)	3 (13.0)	14 (60.9)	
Total	3 (13.0)	17 (73.9)	3 (13.0)	23 (100.0)	
**OAE**					
Failed	2 (8.7)	7 (30.4)	0 (0.0)	9 (39.1)	0.233
Passed	1 (4.3)	10 (43.5)	3 (13.0)	14 (60.9)	
Total	3 (13.0)	17 (73.9)	3 (13.0)	23 (100.0)	
**Audiometry**					
Failed	2 (8.7)	7 (30.4)	0 (0.0)	9 (39.1)	0.233
Passed	1 (4.3)	10 (43.5)	3 (13.0)	14 (60.9)	
Total	3 (13.0)	17 (73.9)	3 (13.0)	23 (100.0)	
**Referral**					
No, normal examination results	8 (8.9)	34 (37.8)	5 (5.6)	47 (52.2)	0.577
Yes, abnormal examination results	11 (12.2)	27 (30.0)	5 (5.6)	43 (47.8)	
Total	19 (21.1)	61 (67.8)	10 (11.1)	90 (100.0)	
**Diagnosis**					
Abnormal	2 (8.7)	7 (30.4)	0 (0.0)	9 (39.1)	0.233
Normal	1 (4.3)	10 (43.5)	3 (13.0)	14 (60.9)	
Total	3 (13.0)	17 (73.9)	3 (13.0)	23* (100.0)	
					

Pearson’s chi-square test; *Number of children whose census region was found.

**Caption:** N = number of individuals (varies according to the characteristics of the variable); OAE = otoacoustic emissions.

**Figure 3:**
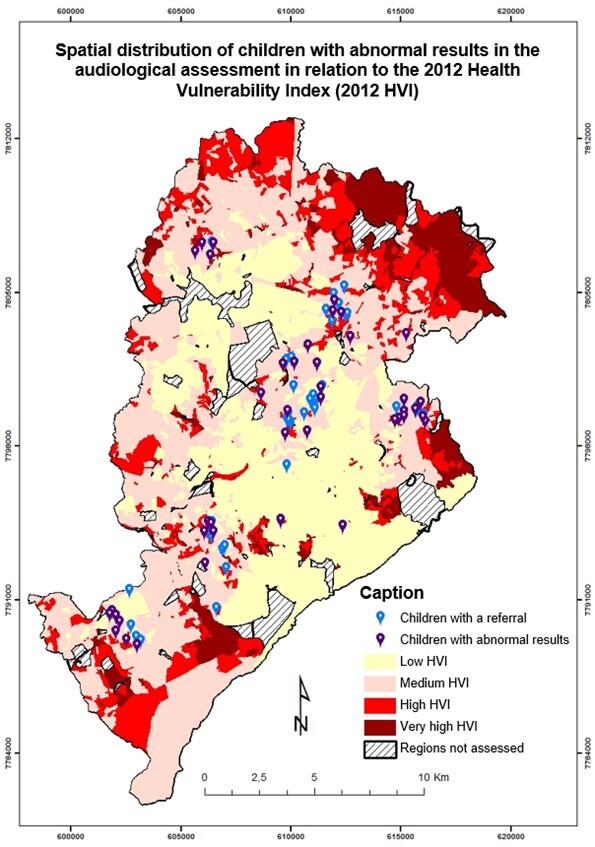
Map of the spatial distribution of children who “failed” Stage 1 and had an abnormal result in Stage 2

The association analysis between HVI and screening results (“pass” and “fail”) found no statistically significant values. However, it was verified that 8.9% of the children who “passed” it (normal examination results) lived in low-risk census regions; 37.8%, in medium-risk regions; and 5.6% in high/very high-risk regions. Of those who “passed” the screening, 12.2% lived in low-risk census regions; 30.0%, in medium-risk regions; and 5.6%, in high/very high-risk regions. The association analysis between HVI and diagnosis likewise did not find statistically significant values. Of the children with a diagnosis of change, 77.7% were from medium-risk census regions. The association analysis between HVI and the results of stages one and two did not find statistically significant values. Of the total 22 children who did not attend the diagnostic stage, 17 (77%) lived in low and medium-risk census regions, although the association was not statistically significant (p = 0.306) (Figure 3).

## DISCUSSION

The analyses in this study showed that almost half of the children screened at the day care centers “failed” it and had to be referred to the diagnostic stage. However, little more than half of them attended the assessment. The prevalence of conductive hearing loss in the assessed sample was 13.7%.

Even though the literature discusses hearing screening in students in terms of protocolization and purpose^(14,15)^, the present study has an unprecedented approach, aiming to broaden the discussion on factors associated with hearing loss intervention results and strategies for this age range.

Thus, the age addressed in this study was intentionally defined at 25 to 36 months, given their language and hearing development process and enrolment in preschool. Also, this is the age that connects studies on NHS^(7)^ and the assessment of students^(14)^. According to the recommendations of the Commission for the Early Detection of Childhood Deafness (CODEPEH) regarding early diagnosis, the period from 0 to 6 years old requires greater attention to prevent and promote health, and it is when hearing must be periodically assessed^(16)^.

In this context, a longitudinal study in 35,668 children that had been submitted to NHS and retested after first grade verified that 3.65 per 1,000 children had permanent hearing loss. The prevalence of moderate to profound bilateral hearing loss was 1.51 per 1,000. However, the NHS had identified only 0.9 children with this degree of hearing loss per 1,000^(17)^.

The prevalence of conductive hearing loss verified in this study is not surprising, as the literature points out that otitis media is the most common cause of hearing loss in children aged 1 to 5 years^(5,18-20)^. Thus, hearing screening is extremely important in the school context, as the population in this age range is more vulnerable to diseases (including otitis media) because of their still developing immune system.

Another information that corroborates the literature concerning the occurrence of conductive loss^(5,18-20)^ is that almost half of the sample failed examinations, and about 40.0% failed tympanometry (screening) with types B and C tympanograms at the time of the assessment. This ‘’moment” of hearing deprivation can cause central auditory processing disorders, phonetic and phonological deviations, and learning, reading, and writing difficulties^(3,18)^.

It can be verified that the literature on the topic has various results on the prevalence of conductive hearing loss in studies involving hearing screening. A study conducted in Rio de Janeiro assessed 431 children aged 1 to 12 years^(5)^ and indicated that 24.12% of the assessed sample had middle-ear changes, and the most common abnormal tympanograms were types B and C^(16)^. Also, a study conducted in inland Malawi, in West Africa, in 281 children aged 4 to 6 years indicated conductive loss in 46.9% of the sample^(21)^. The varying results may be ascribed to factors such as sample definition, given that the age range may be related to conductive loss susceptibility or assessment seasonality, as conductive losses are more common in certain climate conditions.

The occurrence of almost 15% of OAE “fail” results in this study’s children who had “passed” the tympanometry suggests that these cases have hearing thresholds above 30 dBHL^(5)^. A study conducted at a reference NHS service of a University Hospital assessed 261 newborns with risk factors for hearing loss and verified that 13.40% of them “failed” TEOAE due to temporary conductive loss, identified with tympanometry and otorhinolaryngological assessments^(22)^. Thus, the results reinforce the importance of using this procedure and corroborates the literature that indicates that performing TEOAE timely can help reach a hearing loss diagnosis earlier and define further assessments and the beginning of interventions, such as hearing aid fitting and hearing rehabilitation^(2)^.

When relating different examinations, it must also be pointed out that more than 15% of the children who “passed” OAE did not let professionals perform audiometry on them. This corroborates research in 200 children aged 2 to 5 years, whose comparison analysis between these examinations indicated that about 12% of them did not undergo audiometry for the lack of effective conditioning or impossibility of placing the earphones (child’s refusal), whereas only 2% of participants did not accept the TEOAE probe. Therefore, the researchers concluded that audiometry is not indicated for preschoolers’ assessment^(23)^. Nonetheless, other studies highlight that audiometry is more sensitive than TEOAE, although both can be used to screen preschoolers and schoolers^(24)^.

The present study reinforces the question and broadens the discussion on preschoolers’ hearing screening protocol. The statistical significance between second-stage TEOAE and tympanometry and audiometry results also demonstrates the importance and appropriateness of OAE as a school screening procedure.

Constructing the method in two stages (screening and diagnosis) is adequate and mentioned in the literature^8^. However, the present study had the adherence of only one-fourth of the sample referred for diagnosis at a specialized service in the health system - which may have compromised the final result. This finding corroborates a study in preschools in the same municipality, which likewise had low adherence to the diagnostic stage^(15)^.

In a recent literature review, the studies report adherences to diagnosis ranging from 10% to 65%. The potential reasons they cite for low adherence include the impossibility to contact parents and report the results, the parents’ lack of knowledge of the medical meaning of hearing loss, the cost of subsequent care, the parents’ unavailability to be absent at work, and geographical barriers. In general, there was an almost unanimous recommendation that this aspect is crucial to increase the overall effectiveness of school hearing screening programs worldwide^(25)^.

The final result of more than one-third of the children who attended diagnosis was conductive hearing loss. This information converges with a similar study conducted in 87 Chilean children aged 3 to 5 years, in which 15% of the sample had conductive hearing loss^(18)^. Other similar studies^(5,26-28)^ also showed that conductive loss was the most recurrent among preschoolers.

Concerning the third stage of the study (health vulnerability analysis), most participating children (almost two-thirds of the sample) lived in medium-risk census regions, and less than 10% lived in high/very high-risk regions. These data do not corroborate the HVI distribution published in 2018^(29)^ by the municipal government, which indicated that less than 40% of the population lived in medium-risk census regions, and a little more than one-fourth lived in high/very high-risk regions. Two issues may explain this. The first one is the selection of one day care center per administrative region, so that the sample distribution did not correspond to the population scenario in the municipality. The second explanation is the institutions’ profiles, which are day care centers partnered with the municipal government.

The HVI vulnerability analysis shows that the apparent similarity in the profile of children who “failed” the first and second stages may result from the greater concentration of participants in medium-risk census regions. Hence, the greater concentration of changes in this stratum must be cautiously analyzed, verifying it based on the distribution of participants in each stratum. Nevertheless, it must be considered that the 7.8% loss at georeferencing may refer to addresses in urban agglomerations, which would correspond to very-high HVI areas. Research in children who lived in the same municipality of this study and were assessed at a reference NHS service in 2010 and 2011 showed that 46.6% of the children lived in high or very high-risk census regions, and there was a greater proportion of “fail” results in children who lived in areas of greater health vulnerability^(30)^.

Even though the present study did not find statistical significance in the association between HVI and sex and auditory examination, there is some similarity with the abovementioned study. The proportion of children who “failed” the first stage and lived in low-risk census regions was smaller than those who “failed” it and lived in medium-risk regions.

Lastly, it is important to highlight the hearing screening approach used in this study. Research on this topic usually studies it from a clinical-assistance or prevention perspective^(31)^. The methodology used in this study makes progress in addressing social health determinants regarding hearing diagnoses and, therefore, proposing strategies. Thus, it is clearly important to approach the hearing function in integration with life and discuss to what extent vulnerability can hinder prevention, monitoring, and diagnostic actions.

## CONCLUSION

This study found no statistically significant association between hearing loss and HVI. However, it was found that 77.7% of the children diagnosed with hearing loss lived in HVI middle-risk census regions.
