# ERRATUM: Prevalence of hearing loss and health vulnerability in children aged 25 to 36 months: an analysis of spatial distribution

**DOI:** 10.1590/2317-1782/20242023317en

**Published:** 2024-12-13

Due to an honest mistake by the authors, the article “Prevalence of hearing loss and health vulnerability in children aged 25 to 36 months: an analysis of spatial distribution” (DOI https://doi.org/10.1590/2317-1782/20232021189en), present in CoDAS 2023;35(6):e20210189, was published with a typographical error in the name of one of the authors.

On page 1, **where the text reads:**

“Artur Marins Moreto Silva”

**It should read:**

“Artur Martins Moreto Silva”

The authors apologize for the error.

