# Supplementary material for: Prevalence of hearing loss and health vulnerability in children aged 25 to 36 months: an analysis of spatial distribution
Source: Codas. 2023 Dec 4;35(6):e20210189. doi: 10.1590/2317-1782/20232021189en (PMC10723582; doi:10.1590/2317-1782/20232021189en)
Supplement: Translation

# Translation: Prevalência de perda auditiva e vulnerabilidade a saúde em crianças de 25 a 36 meses: uma análise da distribuição espacial

**DOI:** 10.1590/2317-1782/20232021189pt

**Authors:** Aline Aparecida Lopes, Amélia Augusta de Lima Friche, Stela Maris Aguiar Lemos, Lorena Bicalho, Artur Marins Moreto Silva, Thamara Suzi dos Santos, Renata Cristina Cordeiro Diniz Oliveira, Paul Avan, Sirley Alves da Silva Carvalho

**Affiliations:** 1 Programa de Pós-graduação em Ciências Fonoaudiológicas, Universidade Federal de Minas Gerais – UFMG - Belo Horizonte (MG), Brasil.; 2 Departamento de Fonoaudiologia, Faculdade de Medicina, Universidade Federal de Minas Gerais – UFMG - Belo Horizonte (MG), Brasil.; 3 Programa de Pós-graduação em Ciências da Saúde da Criança e do Adolescente, Universidade Federal de Minas Gerais – UFMG - Belo Horizonte (MG), Brasil.; 4 Instituto de Geociências, Universidade Federal de Minas Gerais – UFMG - Belo Horizonte (MG), Brasil.; 5 Laboratoire de Biophysique Neurosensorielle, Université Clermont-Auvergne, France.; 6 Institut de l'Audition de l'Institut Pasteur – INSERM - Paris, France.

**Keywords:** Saúde da Criança, Perda Auditiva, Vulnerabilidade em Saúde, Pré-Escolar

## Abstract

**Objetivo:**

Analisar a associação entre perda auditiva e a vulnerabilidade à saúde em crianças na faixa etária de 25 a 36 meses.

**Método:**

Estudo observacional analítico do tipo transversal realizado por meio da triagem auditiva infantil em nove creches. A triagem constou de anamnese, meatoscopia, timpanometria, emissões otoacústicas transientes e audiometria tonal limiar. Para cada exame realizado foi estabelecido o critério de “passa” e “falha”. Os endereços residenciais das crianças foram georreferenciados e foi construído mapa coroplético da distribuição espacial, considerando o Índice de Vulnerabilidade à Saúde (IVS). Foi realizada análise de associação entre o IVS com as variáveis sexo, exames audiológicos e regional de domicílio por meio dos testes Qui-quadrado de Pearson, e Exato de Fisher.

**Resultados:**

Foram avaliadas 95 crianças de ambos os sexos, destas, 44,7% apresentaram alteração em pelo menos um dos exames realizados, sendo encaminhadas para avaliação otorrinolaringológica e auditiva. Das alterações observadas 36,9% ocorreram na timpanometria e 7,8% nas emissões otoacústicas transientes. Dentre crianças encaminhadas para avaliação, 9,7% apresentaram diagnóstico de perda auditiva do tipo condutiva, 13,6% resultados dentro da normalidade e 21,4% não compareceram para reavaliação. Das crianças que apresentaram o diagnóstico final de perda auditiva do tipo condutiva (9,7%), 1,9% foi classificado como IVS de risco baixo e 6,8% como IVS de risco médio. Houve significância estatística entre IVS e o local de residência da criança.

**Conclusão:**

Não houve associação com significância estatística entre alteração auditiva e IVS, entretanto foi possível observar que 77,7% das crianças com diagnóstico de perda auditiva residiam em setores censitários de risco médio do IVS.

## INTRODUÇÃO

A articulação dos conceitos de audição e vulnerabilidade pode parecer tênue. Contudo, é fundamental a discussão do diagnóstico auditivo sob a luz das condições de vulnerabilidade e saúde, sobretudo em crianças em seus anos iniciais.

É preciso destacar que tal discussão não exclui a compreensão de que os primeiros anos de vida da criança são o período em que ocorre o maior processo de maturação do sistema auditivo central assim como a plasticidade neural da via auditiva^(1-3)^ e, tampouco, da importância do acesso e trajetória no sistema de saúde na garantia da assistência integral e em tempo oportuno.

O processo de identificação e diagnóstico em crianças é complexo e pode ser atravessado por barreiras socioeconômicas, assistenciais e culturais. Estudos mostram que a média de idade em que ocorre a identificação da perda auditiva está em torno de dois anos e meio a três anos de idade, o que já é tardio considerando as recomendações científicas que priorizam a adaptação de dispositivos eletrônicos antes dos seis meses de idade^(3-6)^. Segundo dados de pesquisa prévia mesmo a Triagem Auditiva Neonatal sendo obrigatória, a cobertura na região sudeste atinge apenas 70,3% dos recém-nascidos^(7)^. O estudo mostra que embora no Brasil ocorra uma evolução positiva, os índices ainda são baixos, pois no período de janeiro de 2008 a junho de 2015 a cobertura final foi de 31,8% com variação entre as regiões brasileiras de 19% a 100%, indicando distribuição espacial desigual e as melhores coberturas concentradas nas Regiões Sul e Sudeste^(7)^.

Neste contexto, indubitavelmente, é preciso inserir a discussão sobre a vulnerabilidade e uso dos seus indicadores em estudos de prevalência e diagnóstico em audição. A literatura aponta que o uso desses instrumentos pode facilitar a compreensão de demandas e de informação para formulação de políticas, para a tomada de decisões, ou ainda na divulgação de informações^(8)^. No presente estudo a vulnerabilidade foi considerada sob a perspectiva dos determinantes sociais da saúde sendo estudada com o uso Índice de Vulnerabilidade à Saúde (IVS), desenvolvido pela Secretaria Municipal de Saúde de Belo Horizonte^(9)^ em busca da articulação com o processo de triagem auditiva na faixa etária de 25 a 36 meses.

Sabe-se que o diagnóstico inicial não alcança a totalidade das crianças brasileiras e, portanto, muitas chegam à educação infantil sem uma abordagem adequada. Em conseguinte, a triagem auditiva em escolares inseridos na educação infantil poderá prevenir dificuldades no desenvolvimento da linguagem oral e escrita, já que ambas estão diretamente ligadas à audição. Cerca de 50% das perdas auditivas poderiam ser evitadas ou suas sequelas diminuídas se ocorressem precocemente medidas de identificação, diagnóstico e reabilitação, principalmente em crianças em idade escolar^(6,10)^.

Diante da relevância do diagnóstico da deficiência auditiva em tempo oportuno na infância, seu impacto no desenvolvimento global e na qualidade de vida da criança e a triangulação com os determinantes sociais, este estudo tem como objetivo analisar a associação entre alterações auditivas e a vulnerabilidade à saúde em crianças na faixa etária de 25 a 36 meses matriculados em creches públicas.

## MÉTODOS

Trata-se de estudo observacional analítico do tipo transversal, aprovado pelo Comitê de Ética da Universidade Federal de Minas Gerais sob o número 931.831 e faz parte de um estudo maior intitulado Desenvolvimento de instrumento para triagem auditiva infantil.

No presente estudo a amostra foi composta por crianças na faixa etária de 25 a 36 meses matriculadas em creches conveniadas à prefeitura municipal de Belo Horizonte. O cálculo amostral foi realizado para o projeto maior, no qual foram avaliadas crianças em três diferentes faixas etárias: 12 a 18 meses, 25 a 36 meses e 37 a 48 meses. Entretanto, esta pesquisa avaliou apenas as crianças da faixa etária de 25 a 36 meses de idade. O Cálculo amostral foi realizado para o estudo principal, considerando: os três grupos etários, que a verdadeira taxa de alteração auditiva na população de Belo Horizonte dificilmente exceda 30%; erro de 5%; e nível de significância de 5%. Considerando perda de 10% da população, o cálculo final sugeriu amostra de 108 de cada grupo etário. Porém, no presente estudo, apenas 95 responsáveis de crianças na faixa etária de 25 a 36 meses de idade assinaram o termo de consentimento livre esclarecido (TCLE), o que definiu o tamanho amostral.

Este estudo foi realizado em nove creches da região metropolitana de Belo Horizonte, localizadas em cada uma das nove regionais administrativas da cidade: Barreiro, Centro-Sul, Leste, Nordeste, Noroeste, Norte, Oeste, Pampulha e Venda Nova. Para que fosse obtida representatividade de todo o município de Belo Horizonte, uma creche em cada regional foi selecionada e a direção consultada sobre a adesão. Em cada creche participante, todos os responsáveis pelas crianças na faixa etária do estudo foram convidados a participar.

Os Critérios de inclusão para a participação no estudo foram crianças na faixa etária entre 25 e 36 meses, matriculadas em creches conveniadas à Prefeitura de Municipal de Belo Horizonte cujos pais ou responsáveis legais aceitarem e assinarem o TCLE. Foram excluídas crianças que não compareceram à creche no dia da triagem ou avaliação diagnóstica ou que responsáveis informaram a desistência de participação em alguma etapa do estudo.

O estudo constou de três etapas, sendo a primeira denominada triagem, a segunda diagnóstico e a última georreferenciamento. A triagem foi realizada nas creches e o diagnóstico no Hospital São Geraldo HC/UFMG, para aquelas crianças que ‘’falharam” na primeira etapa. Cada etapa contou com procedimentos específicos.

As avaliações na primeira etapa foram realizadas entre os meses de fevereiro a dezembro de 2017, em dois dias da semana, sendo um no período da manhã e outro no outro no período da tarde. O tempo médio para a realização de toda a avaliação foi entre 10 a 20 minutos por criança. Para classificar os resultados dos exames audiológicos foram utilizados os termos: “passa” e “falha”. Os procedimentos da triagem foram os seguintes:

- Meatoscopia: a inspeção do meato acústico externo foi realizada com o Otoscópio Pocket Junior com iluminação por fibra ótica 2,5V 22840 – Welch Allyn.
- Timpanometria: realizada com o imitanciômetro Madsen Otoflex 100, calibrado segundo o padrão ANSI S3.6 com o objetivo de avaliar a integridade do sistema tímpano-ossicular, por meio da curva timpanométrica e pesquisa dos reflexos acústicos ipsi e contralaterais, de todas as crianças avaliadas na meatoscopia. Os resultados da timpanometria foram analisados segundo o padrão de normalidade sugerido por Jerger e Jerger^(11)^
- Emissões Otoacústicas Transientes (EOAT): realizada em cabina acústica portátil modelo mini com dimensões de 90x90x155cm. O aparelho utilizado para o registro das EOAT foi o Elios® da marca ECHODIA. O protocolo de registro adotado, modo screening, utilizou estímulos cliques não lineares a uma intensidade de 80 dB NPS e a janela de testagem foi de 12 milissegundos, com 512 estímulos. As EOAT foram consideradas presentes quando a reprodutibilidade for igual ou maior a 70% e a relação S/R (sinal/ruído), igual ou maior a 3dB foi utilizado o critério ‘’passa” e ‘’falha”. As crianças que apresentaram resultado ‘’falha”, uni ou bilateralmente, foram encaminhadas para avaliação otorrinolaringológica e fonoaudiológica.
  ● Audiometria tonal liminar: realizada em cabina acústica portátil modelo mini com dimensões de 90x90x155cm com o aparelho utilizado Elios® da marca ECHODIA, com uso de fones supra-aurais TDH. Foi realizada pesquisa de limiares de Via Aérea (técnica de varredura em 20 dB), nas frequências: 500, 1000, 2000 e 4000 Hz.^)^ Sempre foram utilizados recursos lúdicos para o condicionamento da criança. As crianças que apresentaram limiares auditivos acima de 20dB NA em pelo menos uma das frequências testadas, ‘’falha”, foram encaminhadas para avaliação otorrinolaringológica e fonoaudiológica.

Vale destacar que os pais receberam um documento de devolutiva contendo os exames realizados e os resultados. No caso das crianças que apresentaram ‘’falha” em algum exame, no mesmo documento constou o agendamento para avaliação otorrinolaringológica e fonoaudiológica para conclusão diagnóstica, no serviço de Audiologia

A segunda etapa, ou seja, a avaliação diagnóstica das crianças que “falharam” em algum dos exames realizados na creche foi realizada no Ambulatório São Geraldo HC/UFMG. A avaliação diagnóstica constou dos seguintes procedimentos: avaliação otorrinolaringológica, timpanometria, EOAT, audiometria tonal condicionada e Potencial Evocado Auditivo de Tronco Encefálico (PEATE). Constaram da equipe de avaliação uma médica otorrinolaringologista, duas fonoaudiólogas e duas estagiárias alunas da graduação em Fonoaudiologia que realizaram os seguintes procedimentos:

- Avaliação Otorrinolaringológica: Foi realizada avaliação otorrinolaringológica completa com remoção de cerúmen com água morna ou cureta quando necessário. Nos casos de infecção aguda de vias aéreas superiores foi realizado tratamento com posterior encaminhamento para as Unidades Básicas de Saúde - UBS para continuar o acompanhamento com otorrinolaringologista ambulatorial. Casos crônicos foram orientados e também encaminhados à UBS.
- Imitanciometria: O aparelho utilizado para o diagnóstico foi o Impedanciômetro modelo AT 235 da marca Interacoustics, calibrado em 23 de agosto de 2017 certificado 4251/2017. Foram pesquisados os reflexos acústicos ipsi e contralaterais nas frequências de 1,2 e 4 kHz. Os resultados da timpanometria foram analisados segundo o padrão de normalidade sugerido por Jerger e Jerger^(11).^
- Audiometria tonal liminar e Logoaudiometria: O aparelho utilizado foi o Audiômetro AD229b da marca Interacoustics, calibrado em 23 de agosto de 2017 certificado 4246/2017. Técnica descendente nas frequências de 250, 500, 1000, 2000, 3000, 4000 e 8000Hz, por via aérea e por via óssea nas frequências de 500, 1000, 2000, 3000 e 4000Hz, logoaudiometria por meio ordem simples e mascaramento auditivo quando necessário. Os resultados foram analisados segundo o padrão de normalidade proposto por BIAP^(12)^
- PEATE: O aparelho utilizado para o registro do PEATE foi o Elios® da marca ECHODIA. Foram pesquisados a integridade de vias auditivas e de limiar eletrofisiológico. O protocolo utilizado foi: estímulo clique rarefeito, filtro passa-baixo 3000 Hz e passa-alto 50 Hz com 17 cliques por segundo e número mínimo de 1000 aquisições. Os eletrodos foram posicionados em Cz, Fz, A1 e A2 e o estímulo foi apresentado por meio do fone de inserção. As impedâncias foram mantidas com limite máximo até 5Kohms. A integridade das vias auditivas foi avaliada em duas varreduras na intensidade de 80dBnNA, por meio da pesquisa das latências das ondas I, III e V, os intervalos interpicos assim como a reprodutibilidade. O limiar eletrofisiológico foi determinado na última intensidade onde ocorreu a última aparição da onda V.
  ● EOAT: realizada com o equipamento Elios® da marca ECHODIA com estímulos cliques não lineares a uma intensidade de 80 dB NPS e a janela de testagem foi de 12 milissegundos, com 512 estímulos. As EOAT foram consideradas presentes quando a reprodutibilidade for igual ou maior a 70% e a relação S/R (sinal/ruído), igual ou maior a 3dB.

Quanto ao fluxo de atendimento na segunda etapa, vale esclarecer que após a avaliação e conduta otorrinolaringológica, as crianças foram encaminhadas para a realização da Imitanciometria e na sequência a audiometria e EOAT. As crianças que apresentaram todos os exames dentro dos padrões de normalidade tiveram resultado: Audição normal. As crianças que não puderam ser condicionadas para a realização da audiometria foram encaminhadas para a realização do exame objetivo - PEATE. Ao final da avaliação foram entregues aos pais ou aos responsáveis os resultados dos exames realizados, e quando necessário foram realizados encaminhamentos para a unidade básica de saúde de referência da família para realização do cadastro da criança no Serviço de Saúde Auditiva.

Por fim, foi realizado o tratamento e análise dos dados. A busca dos endereços correspondentes à residência das crianças avaliadas foi realizada e teve como objetivo a identificação do setor censitário e do IVS, desenvolvido e utilizado pela Secretaria Municipal da Saúde de Belo Horizonte para organização dos serviços de saúde no município. O IVS é um indicador composto, que utilizou dados do Censo de 2010^(13)^, e, por meio de variáveis socioeconômicas e de saneamento, analisa as características de grupos populacionais que vivem em setores censitários. O índice é composto por variáveis referentes aos domicílios particulares permanentes com abastecimento de água, esgotamento sanitário e destino do lixo inadequados ou ausentes; a razão de moradores por domicílio; o percentual de pessoas analfabetas; o percentual de domicílios particulares com rendimento per capita até 1/2 salário mínimo; o rendimento nominal mensal médio das pessoas responsáveis; e o percentual de pessoas de raça/cor parda, preta ou indígena. Ao final do processo, o IVS é classificado em quatro categorias, baixa, média, elevada e muito elevada vulnerabilidade à saúde. No presente estudo foi realizada a identificação do risco correspondente aos setores censitários em que residia cada criança avaliada, sendo que para fins de análise, as categorias elevado e muito elevado, agrupadas em uma.

Para análise de dados as variáveis selecionadas para estudo foram: sexo; idade (em meses); regional de residência; resultados dos exames de meatoscopia, timpanometria, emissões otoacústicas e audiometria, na triagem, classificados como “passou” e “falhou”; encaminhamentos realizados (sim e não); resultados avaliação diagnóstica (exames normais e perdas auditivas); e o IVS dos setores censitários de residência das crianças, categorizado em baixo, médio e elevado/muito elevado risco.

O georreferenciamento dos locais de moradia das crianças foi feito com base no endereço de residência de cada criança e em seguida foram identificados os respectivos setores censitários e suas classificações. Para tal, foram utilizados os programas ArcGis versão 10.5 ferramenta ArcMap e o Google Earth Pro versão 7.3.1. Para a localização dos endereços das crianças foi utilizado o Google Earth Pro, do qual foram extraídos os arquivos de extensão .kml para ser georreferenciado sobre o ArcGis e produzir os mapas sobre a base do IVC-2012 – Prefeitura Municipal de Belo Horizonte. Do total de 95 endereços, 94,8% foram processados e georreferenciados, sendo os setores censitários identificados por meio do programa Google Earth. Não foi possível a localização manual de 5 endereços, correspondendo a 5,2% do total de endereços disponíveis.

Para a análise descritiva foram utilizadas distribuição de frequências absolutas e relativas das variáveis categóricas e medidas de tendência central, posição e dispersão da variável idade.

Para as análises de associação, considerou-se duas variáveis resposta: (1) resultado das EOA e (2) IVS. Para a primeira, avaliou-se a associação com os resultados da audiometria e da timpanometria na triagem e na avaliação diagnóstica. Em relação ao IVS, analisou-se a associação com o sexo, resultados dos exames, encaminhamentos e diagnóstico. Para as análises de associação foram utilizados os testes Qui-quadrado de Pearson e Exato de Fisher, sendo consideradas como associações estatisticamente significantes as que apresentaram valor de p ≤ 0,05. Para entrada, processamento e análise dos dados foi utilizado o software SPSS, versão 21.0.

## RESULTADOS

Foram avaliadas 95 crianças pertencentes a nove creches de cada uma das nove regionais de Belo Horizonte. Dos 46 sujeitos encaminhados para o diagnóstico, 26 compareceram. Dos que compareceram, 14 sujeitos apresentaram os resultados dentro da normalidade no final da avaliação, 2 não permitiram ser avaliados, foram submetidos ao PEATE e apresentaram resultado dentro da normalidade e 10 tiveram os resultados alterados. Dos 26 sujeitos avaliados, 10,5% tiveram o resultado/diagnóstico de Perda Auditiva do tipo Condutiva e foram encaminhados para a Unidade Básica de Saúde para realizar o cadastro no Serviço de Atenção à Saúde Auditiva para dar continuidade ao acompanhamento. Os participantes que não compareceram para a Etapa 2 – Diagnóstico, na data em que foi agendado, foram contatados novamente. Após várias tentativas de agendamento, 22 crianças (47,8%) não compareceram (Figura 1, Tabela 1).

**Figura 1:** Fluxograma do protocolo dos procedimentos da pesquisa e distribuição dos participantes

**Tabela 1:** Distribuição da análise descritiva em relação ao sexo e aos exames auditivos

**Variáveis**	**N**	**%**
**Sexo**		
Feminino	58	56,3
Masculino	45	43,7
Total	103	100,0
**Meatoscopia**		
Normal	95	92,2
Não permitiu ser examinado	7	6,8
Não compareceu	1	1,0
Total	103	100,0
**Timpanometria**		
Curva A	57	60
Curva B	36	37,9
Curva C	2	2,1
Total	95	100,0
**Emissões otoacústicas**		
Falhou	8	8,4
Passou	49	51,6
Não se aplica	38	40
Total	95	100,0
**Audiometria**		
Falhou	0	0,0
Passou	34	35,8
Não permitiu ser examinado	15	15,8
Não se aplica	46	48,4
Total	95	100,0

**Legenda:** N = número de indivíduos.

Das 95 crianças incluídas na pesquisa, a maioria (56,3%) era do sexo feminino (Tabela 1) com média de idade de 29,9 meses, mediana 30,0 e desvio padrão 3,5. Em relação aos exames realizados na etapa triagem 92,2% da população estudada não apresentou alteração na meatoscopia e 60% apresentou curva timpanométrica tipo A. Além disso, a maior parte apresentou resultado “passou” no exame de emissões otoacústicas (51,6%) e na audiometria (35,8%) (Tabela 1).

Após a triagem 46 crianças, ou seja, 48,4% da amostra apresentaram resultado “falha” e, portanto, foram encaminhadas para a segunda etapa e destas 22 não compareceram para a realização do diagnóstico, ou seja apenas 52,2% compareceram. Das crianças que compareceram a etapa de avaliação diagnóstica em relação a amostra total, 14,7% tiveram resultados normais em todos os exames realizados (timpanometria, emissões otoacústicas e audiometria) e 10,5% tiveram o resultado ‘alterado’ (Tabela 2).

**Tabela 2:** Distribuição dos resultados na Etapa diagnóstica

Conduta adotada após a Etapa 1	**N**	**%**
**Encaminhamento**		
Não, exames normais	49	51,5
Sim, exames alterados	46	48,4
Total	95	100,0
**Diagnóstico**		
Não compareceram	22	47,8
Exames normais	14	30,4
Perda condutiva	10	21,7
Total	46	100,0
Resultados da Etapa 2 - Diagnóstico		
**Imitanciometria**		
Alterado	10	10,5
Normal	14	14,7
Não permitiu/Não compareceu	22	23,1
Não se aplica	49	51,6
Total	95	100,0
**EOA**		
Alterado	10	10,5
Normal	14	14,7
Não permitiu/Não compareceu	22	23,1
Não se aplica	49	51,6
Total	95	100,0
**Audiometria**		
Alterado	10	10,5
Normal	14	14,7
Não permitiu/Não compareceu	22	23,1
Não se aplica	49	51,6
Total	95	100,0

**Legenda:** N = número de indivíduos

Do total de 95 crianças cujos pais assinaram o TCLE 12 crianças residiam na regional Barreiro, 10 na Centro Sul, 16 na Leste, 14 na Nordeste, 10 na Noroeste, 14 na Oeste, 10 na Pampulha e 4 na regional Venda Nova. Em relação ao IVS foi observado que 59,2% das crianças participantes residiam em setores censitários de médio risco e 9,7% em elevado/muito elevado risco. Não foi possível a identificação em 4,9% dos participantes por não residirem em Belo Horizonte (Figura 2).

**Figura 2:** Mapa da distribuição espacial das crianças submetidas a avalição audiológica o IVS -2012

Das crianças que falharam na timpanometria, realizada na primeira etapa (triagem) 10,0% residem em setores censitários de baixo risco; 24,4% de médio risco e 4,4% em setores de elevado/muito elevado risco de adoecer e morrer. Das que falharam nas EOA, também realizada nas creches, 3,6% residem em setores censitários de baixo risco, 9,1% de médio risco e 1,8% em setores de elevado/muito elevado risco.

A análise de associação entre o resultado das emissões otoacústicas transientes da segunda etapa com os exames de timpanometria e audiometria (Tabela 3) revelaram associação com significância estatística entre as EOA com a timpanometria e com a audiometria (p≤0,001).

**Tabela 3:** Associação entre o resultado do exame de emissões otoacústicas transientes com os demais exames audiológicos no diagnóstico

**Variáveis**	**Emissões Otoacústicas**			**p-valor**
	**Falhou**	**Passou**	**Total**	
	**N (%)**	**N (%)**	**N (%)**	
**Imitanciometria**				
Alterado	10 (41,7)	0 (0,0)	10 (41,7)	≤0,001
Normal	0 (0,0)	14 (58,3)	14 (58,3)	
Total	10 (41,7)	14 (58,3)	24 (100,0)	
**Audiometria**				
Alterado	10 (41,7)	0 (0,0)	10 (41,7)	≤0,001
Normal	0 (0,0)	14 (58,3)	14 (58,3)	
Total	10 (41,7)	14 (58,3)	24 (100,0)	

Teste Exato de Fisher

**Legenda:** N = número de indivíduos.

Para análise da associação entre o IVS e os exames realizados na etapa de triagem foram excluídas as respostas “perda” e “não reside em BH” do georreferenciamento. A análise permitiu observar que não houve associação com significância estatística em quaisquer dos resultados (Tabela 4). Vale destacar que não foi possível realizar análise de associação com as variáveis meatoscopia e audiometria devido a ausência de resposta “falha”.

**Tabela 4:** Associação entre Índice de Vulnerabilidade de Saúde com sexo e exames audiológicos

**Variáveis**	**Índice de Vulnerabilidade de Saúde**				**p-valor**
	**Baixo**	**Médio**	**Elevado/Muito elevado**	**Total**	
	**N (%)**	**N (%)**	**N (%)**	**N (%)**	
**Sexo**					
Feminino	11 (12,2)	36 (40,0)	6 (6,7)	53 (58,9)	0,993
Masculino	8 (8,9)	25 (27,8)	4 (4,4)	37 (41,1)	
Total	19 (21,1)	61 (67,8)	10 (11,1)	90 (100,0)	
**Meatoscopia**					
Alterada	0 (0,0)	0 (0,0)	0 (0,0)	0 (0,0)	----
Normal	19 (21,1)	61 (67,8)	10 (11,1)	90 (100,0)	
Total	19 (21,1)	61 (67,8)	10 (11,1)	90 (100,0)	
**Timpanometria**					
Falhou	9 (10,0)	22 (24,4)	4 (4,4)	35 (38,9)	0,675
Passou	10 (11,1)	39 (43,3)	6 (6,7)	55 (66,1)	
Total	19 (21,1)	61 (67,8)	10 (11,1)	90 (100,0)	
**EOA**					
Falhou	2 (3,6)	5 (9,1)	1 (1,8)	8 (14,5)	0,838
Passou	8 (14,5)	34 (61,8)	5 (9,1)	47 (85,5)	
Total	10 (18,2)	39 (70,9)	6 (10,9)	55 (100,0)	
**Audiometria**					
Falhou	0 (0,0)	0 (0,0)	0 (0,0)	0 (0,0)	----
Passou	6 (18,2)	23 (69,7)	4 (12,1)	33 (100,0)	
Total	6 (18,2)	23 (69,7)	4 (12,1)	33 (100,0)	

Teste Qui-quadrado de Pearson

**Legenda:** N = número de indivíduos (varia devido a característica da variável); EOA= Emissões otoacústica.

Na análise de associação entre o IVS e sexo e exames audiológicos na segunda etapa não houve resultado com significância estatística em quaisquer das análises realizadas (tabela 5).

**Tabela 5:** Associação entre Índice de Vulnerabilidade de Saúde com sexo e exames audiológicos da etapa diagnóstico e conduta

**Variáveis**	**Índice de Vulnerabilidade de Saúde**				**p-valor**
	**Baixo**	**Médio**	**Elevado/Muito elevado**	**Total**	
	**N (%)**	**N (%)**	**N (%)**	**N (%)**	
**Sexo**					
Feminino	11 (12,2)	36 (40,0)	6 (6,7)	53 (58,9)	0,993
Masculino	8 (8,9)	25 (27,8)	4 (4,4)	37 (41,1)	
Total	19 (21,1)	61 (67,8)	10 (11,1)	90 (100,0)	
**Timpanometria**					
Falhou	2 (8,7)	7 (30,4)	0 (0,0)	9 (39,1)	0,233
Passou	1 (4,3)	10 (43,5)	3 (13,0)	14 (60,9)	
Total	3 (13,0)	17 (73,9)	3 (13,0)	23 (100,0)	
**EOA**					
Falhou	2 (8,7)	7 (30,4)	0 (0,0)	9 (39,1)	0,233
Passou	1 (4,3)	10 (43,5)	3 (13,0)	14 (60,9)	
Total	3 (13,0)	17 (73,9)	3 (13,0)	23 (100,0)	
**Audiometria**					
Falhou	2 (8,7)	7 (30,4)	0 (0,0)	9 (39,1)	0,233
Passou	1 (4,3)	10 (43,5)	3 (13,0)	14 (60,9)	
Total	3 (13,0)	17 (73,9)	3 (13,0)	23 (100,0)	
**Encaminhamento**					
Não, exames normais	8 (8,9)	34 (37,8)	5 (5,6)	47 (52,2)	0,577
Sim, exames alterados	11 (12,2)	27 (30,0)	5 (5,6)	43 (47,8)	
Total	19 (21,1)	61 (67,8)	10 (11,1)	90 (100,0)	
**Diagnóstico**					
Alterado	2 (8,7)	7 (30,4)	0 (0,0)	9 (39,1)	0,233
Normal	1 (4,3)	10 (43,5)	3 (13,0)	14 (60,9)	
Total	3 (13,0)	17 (73,9)	3 (13,0)	23* (100,0)	

Teste Qui-quadrado de Pearson

* Número de crianças cujo setor censitário foi encontrado.

**Legenda:** N = número de indivíduos (varia devido a característica da variável); EOA= Emissões otoacústica.

Na análise de associação entre o IVS e os resultados da triagem –“passa” e “falha”- não foram observados valores com significância estatística. Entretanto, verificou-se que entre as crianças que tiveram os resultados ‘’passa” (exames dentro dos padrões de normalidade), 8,9% delas residem em setores censitários de baixo risco, 37,8% em setores de médio risco e 5,6% em setores de risco Elevado/Muito elevado. Das crianças que apresentaram resultado ‘’falha”, 12,2% residiam em setores censitários de baixo risco, 30,0% em setores de risco Médio e 5,6% em setores de risco Elevado/Muito elevado. Na análise de associação entre o IVS e o diagnóstico também não foram observados valores com significância estatística. Das crianças que apresentaram diagnóstico alterado 77,7% são provenientes de setores censitários de médio risco. As análises de associação entre o IVS e resultados da etapa um e dois não revelaram resultados com significância estatística. Do total de 22 crianças que não compareceram à etapa diagnóstica, 17 (77%) residem em setor censitário de risco baixo e médio, contudo a associação não foi estatisticamente significativa (p= 0,306) (Figura 3).

**Figura 3:** Mapa da distribuição espacial das crianças com resultado ‘’falha”na Etapa 1 e com resultado alterado na Etapa 2

## DISCUSSÃO

As análises do presente estudo revelaram que quase metade das crianças triadas nas creches apresentaram resultado “falha” e necessitaram de encaminhamento para a etapa de diagnóstico, contudo, pouco mais da metade compareceu. A prevalência da alteração auditiva condutiva, na amostra avaliada, correspondeu a 13,7%.

Embora a literatura discuta a triagem auditiva em escolares na perspectiva da protocolização e da finalidade^(14,15),^ a abordagem apresentada no presente estudo é inédita e tem como objetivo ampliar a discussão de fatores associados a resultados e estratégias de intervenção de perdas auditivas nessa faixa etária.

Deste modo, a definição da faixa etária, entre 25 e 36 meses, para o estudo foi intencional, devido ao processo de desenvolvimento de linguagem e audição e da etapa de ingresso na educação infantil. Além disso, é a idade de ligação entre estudos que envolvem a triagem auditiva neonatal^(7)^ e avaliação de escolares^(14).^ Segundo as recomendações da Comissão para a detecção precoce da surdez na infância (CODEPEH), em relação ao diagnóstico precoce, o período dos zero aos seis anos demanda maior atenção para a prevenção e promoção da saúde, quando as avaliações audiológicas devem ser realizadas periodicamente^(16)^.

Neste contexto, um estudo longitudinal com 35.668 crianças que foram submetidas a triagem auditiva neonatal e testadas novamente após o primeiro ano da escola primária constatou que 3,65 a cada 1.000 crianças tinham perda auditiva permanente. A prevalência de perda auditiva bilateral de grau moderado a profundo foi de 1,51 em 1.000. Entretanto, apenas 0,9 a cada 1.000 com este grau de perda auditiva tinha sido identificada pela triagem auditiva neonatal^(17)^.

A prevalência de perda auditiva condutiva verificada no presente estudo não surpreende, visto que a literatura aponta a otite média como a causa mais comum de perda auditiva em crianças na faixa etária de 1 a 5 anos^(5,18-20) .^ Assim, a triagem auditiva é de extrema importância no contexto escolar porque a população nessa faixa etária é mais vulnerável a doenças devido ao seu sistema imunológico ainda estar em desenvolvimento, inclusive a otite média.

Outro dado que corrobora a literatura quanto à ocorrência de alterações condutivas^(5,18-20)^ é o fato de quase metade da amostra, falhar em algum dos exames realizados, sendo que cerca de 40,0% com falha na timpanometria (triagem) com configuração de curvas do tipo B e C no momento da avaliação. Esse ‘’momento” de privação auditiva pode provocar desordens do processamento auditivo central, desvio fonético e fonológico, dificuldade de aprendizagem, leitura e escrita^(3,18)^.

É possível verificar na literatura sobre a temática resultados diversos quanto a prevalência de alterações auditivas condutivas em estudos envolvendo triagem auditiva. Estudo realizado no Rio de Janeiro avaliou 431 crianças na faixa etária de um a 12 anos^(5)^ indicou que, 24,12% da amostra avaliada tinha alteração de orelha média e os timpanogramas alterados mais comuns foram os do tipo “B” e “C”^(16)^. Já estudo realizado no interior da cidade de Malawi na África Ocidental, com 281 crianças faixa etária de 4 a 6 anos, indicou alteração condutiva em 46,9% da amostra^(21)^. A variação entre resultados pode ser atribuída a fatores tais como definição de amostragem, pois a faixa etária pode estar relacionada a suscetibilidade de alterações condutivas ou a sazonalidade da avaliação, visto que perdas condutivas são mais comuns em determinadas condições climáticas.

No presente estudo a ocorrência de quase 15% do resultado falha nas emissões otoacústicas das crianças que apresentaram resultado passa na timpanometria sugere nestes casos limiares superiores a 30dBNA^(5)^. Em um estudo realizado no Serviço de Referência em Triagem Auditiva Neonatal de um Hospital Universitário que avaliou 261 neonatos com Indicador de Risco para Perda Auditiva (IRDA), foi verificado que 13,40% de resultado falha nas EOAT em decorrência de alteração condutiva temporária identificada por meio da timpanometria e avaliação otorrinolaringológica^(22)^. Deste modo, os resultados reforçam a importância do uso do procedimento e corrobora a literatura que indica que a realização da EOAT em tempo oportuno pode antecipar o diagnóstico da perda auditiva, favorecer o delineamento de outras avaliações e início de intervenções como a adaptação de AASI e reabilitação auditiva^(2)^.

Outro dado que vale ser mencionado ao abordar a triangulação de exames é o fato de mais de 15% das crianças com resultado passa nas emissões otoacústicas não permitirem a realização da audiometria. Esse dado corrobora pesquisa realizada com 200 crianças na faixa etária entre 2 e 5 anos que em uma análise de comparação entre estes exames indicou em torno de 12% não realização da audiometria por falta de condicionamento efetivo, ou impossibilidade de colocação dos fones (recusa da criança) e em, contrapartida, somente 2% dos participantes não permitiram a colocação da sonda para a realização das EOAT. Assim, os pesquisadores concluíram que a audiometria não é um exame indicado para avaliação em pré-escolares^(23)^. Outros estudos, porém, salientam que embora ambas ferramentas possam ser utilizadas na triagem de pré-escolares e escolares, a audiometria apresenta maior sensibilidade quando comparada a EOAT^(24)^.

O presente estudo reforça tal questionamento e amplia a discussão do protocolo de triagem auditiva em pré-escolares. Outro ponto que evidencia a importância e pertinência das emissões otoacústicas como procedimento de triagem escolar foi a significância estatística entre o resultado das emissões otoacústicas transientes da segunda etapa com os exames de timpanometria e audiometria.

Embora a metodologia da construção de duas etapas, triagem e diagnóstico, seja adequada e mencionada pela literatura^8^, no presente estudo a adesão de apenas ¼ da amostra encaminhada para diagnóstico no componente especializado do sistema de saúde pode ter comprometido o resultado final. Este achado corrobora estudo realizado na educação infantil do mesmo município e apresentou baixa adesão à etapa de diagnóstico^(15)^.

Em uma revisão de literatura recente, os estudos relatam uma variação da adesão ao diagnóstico entre 10% e 65%. As razões potenciais citadas para a baixa adesão incluíram a impossibilidade de contatar e notificar os pais sobre os resultados, a falta de conhecimento dos pais sobre o significado médico da perda auditiva, o custo dos cuidados subsequentes, a inabilidade dos pais para faltar ao trabalho e barreiras geográficas. De modo geral, houve uma recomendação quase unânime de que este é um aspecto crucial para aumentar a eficácia geral dos programas de triagem auditiva escolar em todo o mundo^(25)^.

Contudo, das crianças que compareceram, mais de um terço tiveram, como resultado final, perda auditiva do tipo condutiva. Este dado está em convergência com estudo similar realizado no Chile com 87 crianças na faixa etária entre 3 a 5 anos, no qual 15% da amostra apresentaram perda auditiva do tipo condutiva^(18).^ Outros estudos similares^(5,26-28)^ também mostraram que a perda condutiva apresentou maior ocorrência entre pré-escolares.

Quanto à terceira etapa do estudo, a análise da vulnerabilidade à saúde, vale destacar que a maioria das crianças participantes, quase 2/3 da amostra, residiam em setores censitários de risco médio e menos de 10% em setores de elevado/muito elevado risco. Tais dados não corroboram a distribuição do IVS publicada em 2018^(29)^ pela prefeitura do município que indicava menos de 40% da população residente em setores censitários de risco médio e pouco mais de ¼ em setores de elevado/muito elevado. Tal fato pode ser explicado por duas questões. A primeira em decorrência da seleção de uma creche por regional administrativa e, portanto, a distribuição da amostra não correspondente ao cenário populacional do município. E a segunda explicação reside no perfil das instituições, creches conveniadas a prefeitura.

Ao analisar a vulnerabilidade expressa pelo IVS é possível verificar que a aparente similaridade no perfil de crianças com resultado falha na primeira e na segunda etapas pode ser resultado da maior concentração de participantes nos setores censitários de risco médio. Deste modo, a análise de que há maior concentração de alterações neste estrato deve ser realizada com cautela e usando como balizador a distribuição dos participantes em cada estrato. De qualquer modo, vale considerar que os 7,8% de perda no momento do georreferenciamento podem configurar os endereços correspondentes a áreas de aglomerados urbanos e, portanto, correspondentes às áreas de IVS muito elevado. Uma pesquisa realizada com crianças residentes no mesmo munícipio do presente estudo e avaliadas por um Serviço de Referência de Triagem Auditiva Neonatal (SRTAN), nos anos de 2010 e 2011, mostrou que 46,6% das crianças residiam em setores censitários de risco elevado ou muito elevado e maior proporção de “resultado falha” em crianças residentes em áreas de maior vulnerabilidade à saúde^(30)^.

Ainda que o presente estudo não tenha indicado significância estatística na associação do IVS com sexo e os exames auditivos, há alguma similaridade com o estudo supracitado, pois é possível observar que a proporção de crianças que apresentaram resultado falha na primeira etapa - e residiam em setores censitários de baixo risco foi menor do os que apresentaram resultado falha e residiam em setores de médio risco.

Há que se destacar, por fim, a abordagem dada ao processo de triagem auditiva no presente estudo. Dentre as pesquisas na temática, a via de regra é o estudo na perspectiva clínica-assistencial ou de prevenção^(31).^ A metodologia delineada para o presente estudo permite avançar na perspectiva da determinação social da saúde no contexto do diagnóstico auditivo e na, consequente, proposição de estratégias. Assim, fica clara a importância de pensar a função auditiva na integração com a vida e estabelecer a discussão do quanto a vulnerabilidade pode atravessar as ações de prevenção, monitoramento e diagnóstico.

## CONCLUSÃO

No presente estudo não houve associação com significância estatística entre alteração auditiva e IVS, entretanto foi possível observar que 77,7% das crianças com diagnóstico de perda auditiva residiam em setores censitários de risco médio para o Índice de Vulnerabilidade em Saúde.
