# Supplementary material for: ERRATUM: Prevalence of hearing loss and health vulnerability in children aged 25 to 36 months: an analysis of spatial distribution
Source: Codas. 2024 Dec 13;36(6):e20230317. doi: 10.1590/2317-1782/20242023317en (PMC11702970; doi:10.1590/2317-1782/20242023317en)
Supplement: Translation

# Translation: ERRATA: Prevalência de perda auditiva e vulnerabilidade a saúde em crianças de 25 a 36 meses: uma análise da distribuição espacial

**DOI:** 10.1590/2317-1782/20242023317pt

Devido a erro honesto dos autores, o artigo “Prevalência de perda auditiva e vulnerabilidade a saúde em crianças de 25 a 36 meses: uma análise da distribuição espacial” (DOI https://doi.org/10.1590/2317-1782/20232021189pt), presente em CoDAS 2023;35(6):e20210189, foi publicado com um erro de digitação no nome de um dos autores.

Na página 1, **onde se lia:**

“Artur Marins Moreto Silva”

**Leia-se:**

“Artur Martins Moreto Silva”

Os autores pedem desculpas pelo erro.
